# The quantity of food waste in the garbage stream of southern Ontario, Canada households

**DOI:** 10.1371/journal.pone.0198470

**Published:** 2018-06-13

**Authors:** Paul van der Werf, Jamie A. Seabrook, Jason A. Gilliland

**Affiliations:** 1 Human Environments Analysis Laboratory, University of Western Ontario, London, ON, Canada; 2 Department of Geography, Western University, London, ON, Canada; 3 Department of Paediatrics, Western University, London, ON, Canada; 4 School of Food and Nutritional Sciences, Brescia University College, Western University, London, ON, Canada; 5 Department of Epidemiology & Biostatistics, Western University, London, ON, Canada; 6 School of Health Studies, Western University, London, ON, Canada; 7 Lawson Health Research Institute, London, ON, Canada; 8 Children’s Health Research Institute, London, ON, Canada; University of Waikato, NEW ZEALAND

## Abstract

There is little consensus on the amount of worldwide food waste generation because many current estimates are indirect and link back to the same limited primary datasets, with much of the data originating from fieldwork undertaken in the 1970s and 1980s. Direct measurement of waste streams, through waste composition studies, can be used to develop accurate estimates of food waste disposal. In Ontario, Canada, municipalities that undertake household waste composition studies all use a common direct measurement methodology that includes a broad range of waste categories, including food waste. The purpose of this research was to estimate the quantity of food waste disposed, in the garbage stream, by households in southern Ontario, Canada, and determine if this common methodology could be expanded and serve as the basis of a standardized and rigorous household food waste measurement methodology. Household waste composition study data (2012–2015), including a single “food waste” category, were gathered from 9 Ontario municipalities, aggregated and analyzed to develop estimates of food waste in the garbage stream. On average, households disposed 2.40 kg/week of food waste in the garbage, which comprised 35.4% of this waste stream. This does not include any food waste otherwise disposed (e.g., sink) or recycled (e.g., composted). Urban households disposed significantly greater amounts of food waste compared to rural households in the spring (p = 0.01) and summer (p = 0.02). Households with access to a green bin program disposed significantly less food waste than those with no access to a green bin program in the spring (p = 0.03) and summer (p<0.01). The common methodology used to develop these estimates shows promise as the basis of a household food waste measurement methodology. This future methodology would include dividing food waste into avoidable and unavoidable food waste categories, as well as adding subcategories (e.g., avoidable fruits and vegetables).

## Introduction

Given humanity’s biological nature, the procurement, preparation, eating and wasting of food has been a constant feature of our history. This wasting of food represents lost utility and ultimately, inefficiency. It is ironic, however, that food waste and food insecurity co-exist. On the one hand, Gustavsson et al. [1] estimates that one-third and Parfitt et al. [2] up to one-half of annual food production is wasted, while on the other hand up to 795 million people are undernourished globally, including 15 million in developed regions [3]. Reducing the amount of food that becomes waste can help ameliorate this social issue, as well as presenting opportunities to reduce its monetary (e.g., wasting money) and environmental (e.g., greenhouse gas generation) impacts.

The European Commission [4] defines food waste as “waste composed of raw or cooked food materials, and includes food discarded at any time between farm and fork; in households relating to food waste generated before, during or after food preparation such as vegetable peelings, meat trimmings, and spoiled or excess ingredients or prepared food.” WRAP [5] and Beretta et al. [6] sub-categorize food waste into three categories: (1) Avoidable: Edible food that was thrown away because it was no longer wanted.; (2) Possibly avoidable: Food that some people eat but others do not (e.g., apple peels); may be eaten depending on how it is prepared (e.g., potato skins); or that is thrown out due to a specific criterion (e.g., bent carrots); and (3) Unavoidable: Food that is normally not edible (e.g., banana skin, coffee grounds, inedible slaughter house waste). This also includes losses/wastes from harvesting, storage, transportation and processing that are unavoidable with the best available technologies. In this paper, food waste is either avoidable (i.e., food that was edible at one point) or unavoidable (i.e., food that was never edible).

Since the 1970s, a few researchers have investigated food waste disposal and behaviour [7–10], but this did not coalesce beyond these disparate pockets of research. More recently, due perhaps to the juxtaposition of a rapidly growing population and our improved ability to grow food with ongoing food insecurity, there is a developing critical mass of academic research interest and government intervention and policy development related to reducing food waste. Underlying this interest is the recognition that it is essential to have an accurate and precise understanding of food waste generation. Current food waste generation estimates, across the food supply chain of developed countries, vary widely [1, 4, 11–15] ranging from 96kg/capita/year in a single upstate New York State county [16] to 300 kg/capita/year in North America and Oceania [1]. Much of this food waste is generated by consumers/households, with estimates which also vary widely [5, 11, 17–23], ranging from 19 kg/capita/year in a rural area in Austria [22] to 308 kg/capita/year in Canada. [11] The large variability in estimates is a function of geographic differences but also due to the method used to collect food waste data (e.g., waste audits, diary studies and surveys), the scale of measurement (household, city, national average) and whether the estimate includes avoidable and/or unavoidable food waste.

There is some agreement among researchers about the inadequate state of food waste estimates and that further research is required to improve its measurement [2, 24–26]. There are a number of reasons for these current data gaps. An overarching reason is that there is no international standard, with methods “usually rooted and used regionally or nationally” [27], meaning that studies are not very comparable [22]. This is starting to change with the recent development of a food loss and waste accounting and reporting protocol [28]. Secondly, van der Werf and Gilliland [26] found that there is a high degree of variability of food waste quantity estimates across all parts of the food supply chain. They suggested that there are challenges with the veracity and comparability of this data because of the indirect and direct approaches deployed in its measurement and because the scope of food waste, in current research, varies to include avoidable, unavoidable or both of these food waste streams. These data gaps can be overcome through the development and application of methodological improvements to the measurement of food waste [26].

It is the rationalizing and selecting between the indirect and direct measurement approaches that is central to the required methodological improvements. Most simply, as described by Sharma and McBean [29], indirect methods estimate quantities of food (i.e., domestic food production and imported food) by product categories, and then waste quantities are imputed through the use of waste factors (i.e., percent of a product category that is assumed to become waste). Other indirect measurement methods include statistical estimation due to economic activity [30]. Its main advantage is that it is useful for estimates that have a broad geographic scope (e.g., countries) and its key disadvantage is that it does not physically examine any waste streams. A discrediting factor is that many of these indirect estimates originate from fieldwork undertaken in the 1970s and 1980s [2]. The two-fold challenges of collecting data this way is the age of factors used to make these estimates and the fact that no actual food waste was measured to make these estimates. Referring to the indirect collection of food waste data, Brautigam et al. [12] warns: “it has to be recognised that all calculation methods can only be seen as approximations, which barely reflect reality.” Maystre and Viret [31], Rugg [32], Abdulla et al. [11] and van der Werf and Gilliland [26] all recommend that *direct* measurement be used to estimate food waste.

Direct methods are used to collect, sort, weigh and statistically analyze waste samples collected at the point of generation or just prior to disposal. Its advantage is that actual waste streams are being physically examined. Its disadvantages are that it can be costly and vulnerable to demographic bias (i.e., samples collected not representative) [29]. The main approach to the direct measurement of household food waste, typically referred to as waste characterization studies [33, 34] or waste composition studies [18, 35], involves the curbside collection of household waste samples on their waste collection day. The waste samples typically represent a 1-2-week generation period. Collected waste samples are then taken to a location to be sorted and weighed. Waste samples typically include the garbage stream and may also include green bin (i.e., a separate bin to collect food and other organic waste) and blue box (i.e., a separate bin to collect recyclables) streams.

Key strata used to measure household waste include: geographic location, household type (single family, multi-residential households), waste management system (e.g., bagged waste versus automated collection), housing type, urban/rural areas, socio-demographic differences, and season [27, 29, 35–39]. Ideally, representative sampling areas are randomly selected, although constrained for the above noted factors [38]. To date, direct method studies have examined food waste as part of overall waste composition measurement [18, 40, 41], although there are a growing number of studies that focused exclusively on food waste [22, 23, 42, 43].

The focus of this study is to develop a better understanding of how to directly measure household food waste. There has been little research to specifically measure household food waste in the province of Ontario, Canada. However, many southern Ontario (360,000 km^2^; population of approximately 12 million) municipalities routinely undertake household waste composition studies, using a common methodology [44–47], that typically includes “food” as a sorting category. The first objective of this study was to develop an estimate of the amount of food waste disposed, in the garbage stream, by southern Ontario single-family households using 2012–2015 waste composition study results, collected using this common methodology. A second objective was to determine if this methodology could be adapted and expanded as the basis of the suggested “bespoke and statistically sound methodology” [26] to directly measure household food waste. Both of these study objectives were met.

## Material and methods

### Data collection

Twenty-eight single-family household waste composition datasets, from nine different southern Ontario municipalities (with a population of approximately 2.2 million inhabitants), were gathered, aggregated, and analyzed to estimate single family (i.e., detached, or semi-detached homes) food waste disposal, in the garbage stream. The nine municipalities included a range of large and medium urban (e.g., Greater Toronto Area, southwestern and eastern Ontario) and rural (e.g., central, and southwestern Ontario) municipalities. The datasets, generated from 2012–2015, used a common waste composition study methodology, which is described in [44–47]. This methodology was developed in 2002 and with some refinements has been in use since that time.

Each of the 28 datasets consists of waste composition study data from 100 households. Typically, each sample of 100 households was compiled from 10 sampling areas of 10 consecutive homes strategically selected by the respective municipality to function as a representative sample. One municipality was represented by five sampling areas of 20 homes. Therefore, there were a total of 85 sampling areas across the nine municipalities. Each municipality selects their different sampling areas based on factors such as housing type (e.g., older homes, newer homes) and neighbourhood socio-economic status. The sampling areas are spread out over weekly waste collection days and typically 2 to 4 sampling areas are collected per week day. Waste samples are collected from sampling areas on their waste collection day and are intercepted at the curb prior to municipal collection. The samples are taken to a sorting area and are sorted into as many as 120 sorting categories, including a single “food waste” category. The sorted food waste is weighed and documented. Collection and sorting of wastes is undertaken by waste auditors (i.e., companies that provide professional waste composition study services to municipalities). Each waste composition study is repeated twice over two consecutive weeks for the same households. Thus, two weekly data points (i.e., week 1 and week 2) make up the average of each sampling area’s seasonal data point. Waste composition studies are repeated up to 4 times per year (i.e., to encompass each of the four seasons) for the same sampling areas and households.

Three of the nine municipalities (one large urban, one medium urban, and one rural) divided food waste into avoidable and unavoidable streams and included the results from ten (i.e., sub-set of the 28 waste composition studies) two-week seasonal waste composition studies. This sub-set of waste composition studies were also analyzed separately to develop an estimate of avoidable and unavoidable food waste in the disposal stream.

Furthermore, we compiled data on several variables which could potentially influence the estimates of food waste disposal for inclusion as independent variables in statistical models. For each of the samples, we recorded the waste auditor, season of each study (i.e., winter, spring, summer, fall), sampling area type (i.e., urban, or rural), and household access to food waste diversion programs (i.e., green bin program for collecting food wastes at the curb). In addition, estimates of the number of people per household and median household income (Canadian dollars) were compiled for each sample area using data from the 2011 Canadian census [48] at the dissemination area level, which is the smallest areal unit for which Statistics Canada releases demographic data and is a reliable proxy for each sampling area [49].

### Statistical analysis

Data were analyzed using SPSS version 22 (IBM SPSS Statistics for Windows, Version 22.0. Armonk, NY: IBM Corp.). An average of food waste disposal was developed by averaging all weekly data points from each sampling area. Paired data (i.e., from week 1 and week 2) were used to develop seasonal averages. If there was a missing weekly data point from a sampling area (e.g., if a waste sample was collected by the municipal waste contractor before the waste composition study crew arrived on site), then the other weekly data point was not used to develop the seasonal average. This occurred for 8 of 229 paired data points.

Continuous variables are reported as means and standard deviations, whereas categorical variables are summarized as percentages. The independent samples t-test was used to compare differences in means between two groups, and the paired t-test compared mean differences between food waste disposal estimates across the four seasons. The repeated measures analysis of variance (RMANOVA) assessed differences in the mean food waste per season and by whether homes had green bins. The strength and direction of the associations between two continuous variables were measured using the Pearson correlation coefficient. A multiple regression model was used to assess the influence of urban households, access to a green bin program, and number of people per household on disposal of food waste during the spring and summer months. A 2-sided p value <0.05 was considered statistically significant.

## Results

**Fig 1:**
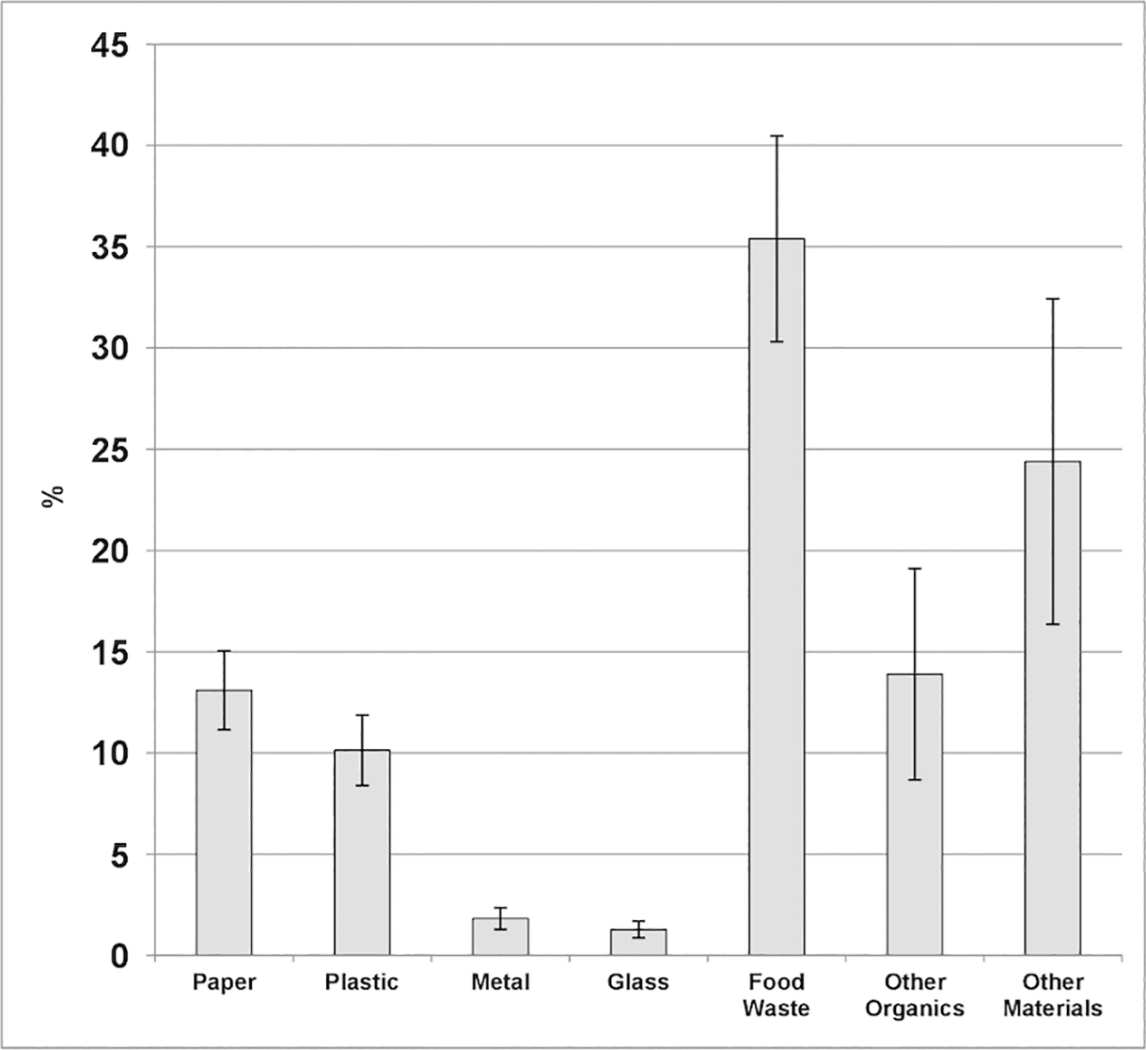
Overall waste composition.

Fig 1 depicts the average waste composition from the 28, two-week single-family household waste composition studies. On average, 35.4% of the disposal (i.e., garbage) stream consisted of food waste (range 27.2%-45.6%). The mean food waste disposal of these households was 2.40 kg/household/week (SD = 1.07) or 124.80 kg/household/year (Table 1). This does not include food waste otherwise disposed (e.g., sink) or recycled (e.g., composted). The range per municipality (n = 9) was 1.78–3.10 kg/ household /week and per waste composition study (n = 28) was 1.41–3.31 kg/ household /week. Furthermore, the per sampling area (n = 85) range was 0.00–4.04 kg/ household /week, with the low part of this range coming from sampling areas with seasonal populations (e.g., summer cottage residents). Variability is based on a neighbourhood basis, but not on a household basis.

**Table 1:** Average weekly food waste disposal for southern Ontario households and impact of waste auditor, sampling area type, access to food waste diversion programs and season.

	n	Mean	SD	p-value
		kg/household/week		
**Food Waste**	85	2.40	1.07	
**Independent Variables**				
**Waste Auditor**				
Waste Auditor 1	35	2.42	1.04	0.91
Waste Auditor 2	50	2.39	1.10	
**Sampling Area Type**				
Rural	50	2.32	0.98	0.37
Urban	35	2.53	1.19	
**Food Waste Diversion Program**				
Green Bin	55	2.28	1.13	0.15
No Green Bin	30	2.63	0.92	
**Season**				
Winter	75	2.33	1.26	
Spring	55	2.30	1.30	
Summer	55	2.39	1.10	
Fall	75	2.36	1.34	

Table 1 summarizes the impact of the independent variables on food waste disposal. There were no differences in food waste disposal as measured by two different waste auditors. Urban households disposed more food waste than rural households; households with access to a green bin program disposed less food waste than households without access to a green bin program; and food waste disposal was marginally higher in the summer and fall. None of these differences, however, were statistically significant.

There was a weak positive correlation (r = 0.29, p = 0.01) between food waste disposal and the number of people living in a household. Weak positive correlations were also found between the number of people in a household and winter disposal (r = 0.21, p = 0.07), spring disposal (r = 0.37, p = 0.01), summer disposal (r = 0.24, p = 0.08), and fall disposal (r = 0.26, p = 0.02). There was no association (r = -0.11, p = 0.34) between food waste disposal and median income, and no relationship between median income and seasonal food waste disposal (r = -0.01 to –0.17).

The relationship between seasonal urban and rural food waste disposal, the impact of having access to a green bin program, and the number of people per household was also assessed. Urban households disposed significantly greater amounts of food waste compared to rural households in the spring (p = 0.01) and summer (p = 0.02) (Table 2).

**Table 2:** Food waste disposal by season and sampling area type.

		n	Mean	SD	p-value
			kg/household/week		
**Winter**	Rural	50	2.25	1.12	0.42
	Urban	25	2.50	1.51	
**Spring**	Rural	40	2.01	1.17	0.01
	Urban	15	3.06	1.36	
**Summer**	Rural	40	2.17	0.99	0.02
	Urban	15	2.96	1.19	
**Fall**	Rural	40	2.22	1.43	0.34
	Urban	35	2.52	1.24	

Finally, using multiple regression models it was determined that in the spring (Table 3) and summer (Table 4), urban households disposed significantly more food waste in both seasons, controlling for the number of people in the household. Furthermore, households with access to a green bin program disposed of significantly less food waste than those with no access to a green bin program in the spring (p = 0.03) and summer (p<0.01).

**Table 3:** Multiple regression on spring food waste disposal (kg/household/week).

	*B*	SE	p-value
**Urban household**	20.11	8.88	0.03
**People per Household**	21.68	12.16	0.08
**Access to a green bin program**	16.23	12.16	0.03
**Constant**	-28.14	34.92	
**Adjusted *R***^***2***^	0.20		

**Table 4:** Multiple regression on summer food waste disposal (kg/household/week).

	*B*	SE	p-value
**Urban household**	22.32	7.25	<0.01
**People per Household**	10.67	9.86	0.28
**Access to a green bin program**	20.41	6.11	<0.01
**Constant**	2.72	28.32	
**Adjusted *R***^***2***^	0.24		

Three of the nine municipalities (one large urban, one medium urban and one rural) divided food waste into avoidable and unavoidable streams and included the results from ten (i.e., sub-set of the 28 datasets) two-week seasonal waste composition studies. Food waste averaged 36.1%. As described in Table 5, avoidable food waste was slightly more than one-half of all food waste.

**Table 5:** Avoidable and unavoidable food waste disposal.

	N	Mean	SD	
		kg/household/week		%
**Avoidable Food Waste**	10	1.3	0.18	52.5
**Unavoidable Food Waste**	10	1.1	0.60	47.5
**Total**	**10**	**2.4**	**0.62**	**100.0**

## Discussion

This study developed an estimate of the amount of food waste disposed, in the garbage stream, by southern Ontario single-family households using a common methodology, and assessed whether this methodology could be adapted and expanded to directly measure household food waste.

This research represents one of the first attempts to use direct at the curb measurement of food waste to measure household food waste in a geographic region, and to examine the influence of various independent variables (e.g., waste auditor, sample area type, food waste diversion program, seasons, number of people per household, and median household income) on the quantity of food waste disposal.

As summarized in van der Werf and Gilliland [26], consumers/households in developed countries dispose 18.8–308.2 kg/capita/year of food waste, with an average of 114.3 kg/capita/year (n = 24; SD = 68.0). In our study, there was an average of 2.9 residents per household and average food waste disposal was 43.0 kg/capita/year. Mindful that our results only encompass food waste disposed in the household garbage stream, this estimate is at the lower end of that range and well below the average. In a recent waste composition study from Guelph, Ontario, it was estimated that households disposed 4.2 kg/capita/week or 217.4 kg/capita of organic waste (i.e., predominantly food waste, and also includes some non-food but compostable items) in their green bin [42]. The amount of food waste in the garbage stream was not included in their estimate. Parizeau et al. [42] estimate is considerably higher than the above noted estimates. It is suggested that the variability between these various estimates is a result of different methodological approaches and actual differences. At this point, the various methodological approaches employed constrain the parsing out of actual differences in household food waste disposal between the results reported in this paper and other studies.

The lack of significant differences between food waste disposal estimates measured by two different waste auditors is an important finding and suggests that this common methodology is reliable and repeatable. The lack of overall seasonal significant differences also suggests that there may be year-round food waste disposal consistency. Additionally, the overall lack of significant differences of food waste disposal, in the garbage stream, between municipalities with and without access to green bin programs is an important finding and suggests that households with access to green bin programs may in fact dispose more food waste than households without this access (i.e. because green bin food waste disposal was not measured). This would need to be confirmed by the future simultaneous measurement of food waste in garbage and green bin disposal streams. While there was no green bin waste composition data for these households, from all Ontario households with access to a green bin program, from 2012–2015 diverted a mean of 2.4kg/household/week (SD = 0.1) [50]. The green bin is primarily for wasted food but also includes non-recyclable paper (e.g. paper towels) and contamination.

The foregoing, however, is tempered somewhat by Spring and Summer findings in which households without green bins disposed of significantly more food waste than households with green bins. Overall, the common methodology employed could form the basis of a more comprehensive household food waste measurement methodology.

Our study is not without limitations. Due to data availability, we only examined food waste disposed in the garbage stream. Our results, therefore, do not encompass any food waste directed to the green bin or informal methods such as backyard composters, feeding to pets, and disposal down the drain. The amount of food waste managed via informal methods can be considerable. For instance, in an Australian study, Reynolds et al. [51] found that households generated a mean of 2.60 kg/week (SD = 2.34) of informal household food waste.

As such, our estimates represent the minimum food waste disposed by households. These partial estimates do, however, address a key household waste stream and can be used to estimate environmental impacts such as greenhouse gases from landfilled food waste. Further, the results only go marginally beyond “food waste” as a waste composition study sorting category and offer little detailed information on the composition of this food waste. Finally, the common methodology is used to collect data at the neighbourhood level (i.e., 10 consecutive households) so we can only be certain of the average of that neighbourhood, but not household-level characteristics of food waste disposal on a house-by-house basis. That is, the common methodology does not measure the variability of food waste generation between individual households. However, to facilitate municipality-level data extrapolation the common methodology includes instructions on how to select up to ten representative sampling areas (i.e., neighbourhoods) [44–47]. Municipalities scale up the results from these neighbourhoods to develop an estimate of the amount of different waste types, including food waste, that go to landfill. Partially assuaging this limitation is that municipalities typically develop interventions on a neighbourhood basis, not at the household level.

Additional research is needed to take this common methodology and use it to develop and test a household food waste measurement methodology that includes avoidable and unavoidable food waste sorting categories, as well as additional food waste sub-categories (e.g., avoidable fruit and vegetables); that measures the food waste in all waste streams (e.g., garbage, green bin); and that is capable of elucidating the impact of the green bin on food waste disposal, and whether or not households with access to green bins dispose more food waste than households without access to green bins. Further, this refined methodology should be expanded to move beyond presenting municipality specific and largely descriptive data and incorporate inferential capabilities, so that it can be used to develop regional and possibly country-wide estimates. This research should consider and build on methodology development undertaken in other jurisdictions. For instance, the UK’s Waste Reduction Action Programme (WRAP) has developed a number of solid and liquid food waste estimates [5, 21, 52, 53] using waste management tonnage data collected by local authorities, the results of waste audits and from kitchen diaries (i.e., food waste tracking by residents). Further, this research demonstrated a possible methodological approach to extrapolating this data. Aspects of the aforementioned research and other approaches have been used in other European and North American countries [20, 22, 42, 54–56] with the focus on using a weight-based assessment of food waste and in some cases including diary studies or surveys.

Understanding food wasting behaviour can inform reduction interventions, and several largely qualitative studies have attempted to develop a better understanding of food wasting behaviours. Food appears to be wasted for various reasons including the pressure to eat properly and provisioning challenges [57]; ingrained household routines leading to a pattern of overprovisioning and inflexibility in meal preparation, which are exacerbated by the sometimes unpredictability of daily life [58]; food safety [59]; lack of planning (for food purchase and preparation) [60]; and social factors (e.g. household type) and intractable consumer food expectations (e.g., freshness, variety) [61]. The direct quantitative estimation of food waste can build on and transcend qualitative data to confirm and track this behaviour.

Bulkeley and Gregson [62] call for greater engagement with households to inform waste policy, arguing that waste policy must “open up the black box that is the household and engage with household practices”. Barr et al. [63] contend that when moving up the waste hierarchy towards reduction means engaging with households “in ways that move beyond the simple disposal of things”. To date, many household food waste estimates have been derived using indirect measurements [26] that do not engage households. Our study builds on other direct household food waste studies, [5, 22, 23, 42, 43, 53, 64] more fully opening the Bulkeley and Gregson [62] “black box” to compare food waste disposal across municipalities based on data collected using the same waste composition study methodology. This, however, is an intermediate step, and consideration should be given to ensuring that the household food waste measurement methodology can be used to measure food waste at the household level, and couple this with direct household interaction to measure the how and why of food wasting behaviour.

## Conclusions

Based on available waste composition study data, households in nine southern Ontario municipalities dispose, on average, 2.4kg/ household /week of food waste in the garbage stream. The common methodology used to develop these estimates shows promise as the basis of a household food waste measurement methodology. Expanding this methodology to encompass greater disposal and composition detail can be used to produce more accurate municipal, regional and possibly country-wide household food waste estimates that can be used to develop sound food waste reduction policy and interventions.

## Supporting information

S1 Table Raw data. (XLSX)
